# Evaluating a possible role for PKG1α redox state in chronic hypoxia-induced pulmonary hypertension

**DOI:** 10.1186/2050-6511-16-S1-A80

**Published:** 2015-09-02

**Authors:** Olena Rudyk, Oleksandra Prysyazhna, Lan Zhao, Philip Eaton

**Affiliations:** 1 BHF Centre of Research Excellence, King's College London, London, SE1 7EH, UK; 2 Experimental Medicine, Imperial College London, London, SW7 2AZ, UK

## Background

Hypoxic pulmonary vasoconstriction is a physiological response enabling efficient oxygen delivery to tissues during transient regional lung hypoxia. However, when larger territories are involved, this adaptive mechanism can be detrimental and chronically lead to maladaptation. Such maladaptive events occur commonly due to high altitude hypoxia or pathologies that cause widespread, chronic pulmonary vasoconstriction resulting in hypoxic pulmonary arterial hypertension (HPAH), maladaptive pulmonary vascular remodelling and right heart failure. Previous work from our lab has shown protein kinase G (PKG) 1α is susceptible to oxidation, forming a disulfide dimer which directly activates the kinase resulting in vasodilation and blood pressure lowering. During hypoxia, pulmonary cells become pro-reducing. In this study we tested the hypothesis that this may alter the levels of oxidant-activated PKG1α and contribute to HPAH pathogenesis.

## Methods

A “redox-dead” Cys42Ser PKGIα knock-in (KI) mouse which cannot be oxidant-activated was employed. HPAH was induced by exposing mice to 10% O_2_ for 4 weeks. Cardiac function and pulmonary arterial stiffness was assessed by echocardiography (VEVO 770, Visual Sonics). Right ventricular pressure was measured using a 1.2F pressure catheter (Scisence Inc). Vascular reactivity was assessed using a tension myograph (DMT). Tissues for molecular analysis were harvested under hypoxic (10% O_2_) or normoxic conditions (room air) respectively to reflect their treatment conditions.

## Results

**Figure 1.**
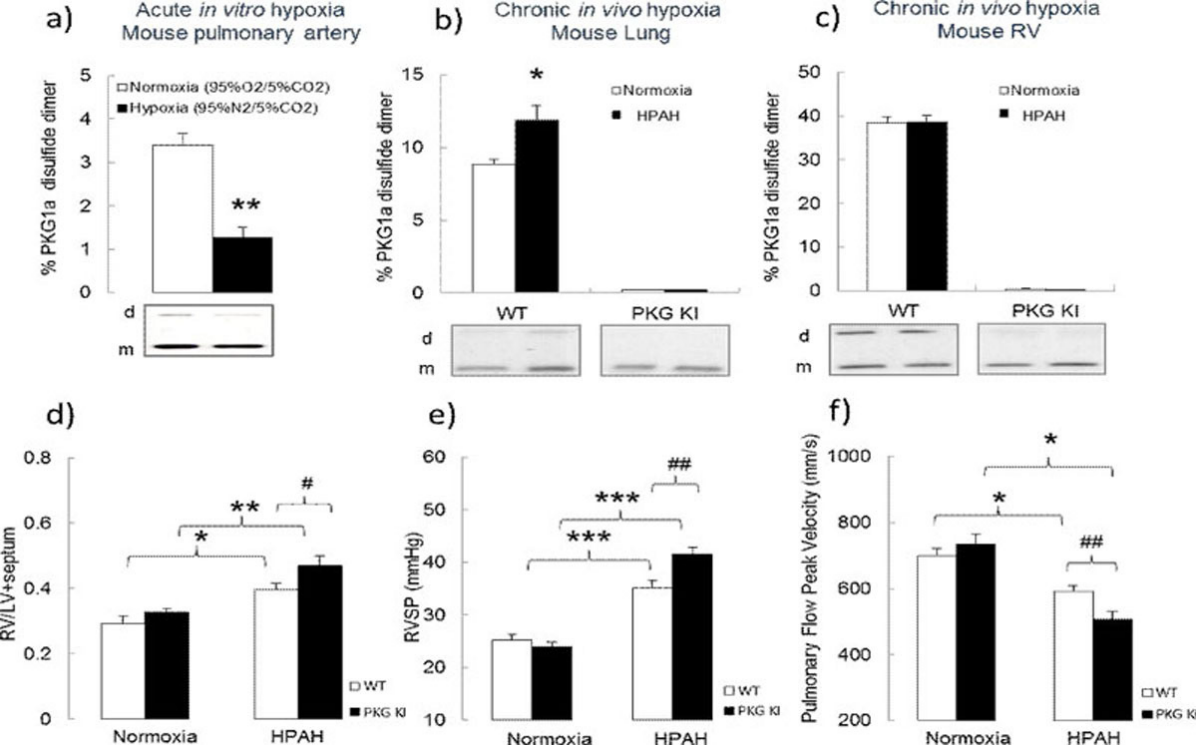
**Differential responses of WT or Cys42Ser PKGIα KI tissues to acute or chronic hypoxia (a)** Decrease in PKG1α disulfide levels in mouse pulmonary artery under acute hypoxia. **(b)** PKG1α disulfide formation in mouse lung and **(c)** right ventricle after chronic hypoxia. **(d)** Fulton index, **(e)** Right ventricle systolic pressure and **(f)** pulmonary flow velocity in KI mice compared with WT mice after HPAH. *p≤0.05, **p≤0.01, ***p≤0.001, by t-test. n=8-10/group.

Acute *in vitro* hypoxia (60 min) resulted in a reduction of PKG1α disulfide level in pulmonary artery (Fig. 1a). A similar decrease in PKG1α disulfide levels was observed in the lung tissue from mice exposed to acute hypoxia (5-20min at 10% O_2_). In contrast, HPAH induced by chronic *in vivo* hypoxia resulted in significant increase of PKG1α disulfide in the lung tissue of WT mice, while the level of PKG1α disulfide in the right ventricle remained similar (Fig 1b-c). There was a trend towards an increase in total PKG1α expression in the lung and right ventricle. After exposure to chronic hypoxia all mice developed HPAH, mild left ventricular dysfunction and mild body weight loss. However, the KI mice developed a more severe HPAH phenotype, i.e. potentiated right ventricular hypertrophy, higher right ventricular systolic pressure and pulmonary arterial stiffness, compared to WT littermates (Fig. 1d-f). Despite these differential changes, both genotypes had similar compensatory increases in haematocrit and blood haemoglobin levels. Total lung weight was significantly increased after chronic hypoxia; however there were no differences between WT and KI mice. In addition, there was no pronounced development of lung oedema in either genotype. *To provide insight into the potential causative* importance of PKG1α redox state in the control of pulmonary vasotone, *we compared* responses of WT and KI isolated pulmonary arteries to H_2_O_2_. Under normoxic conditions, KI pulmonary vessels had a small but significant decrease in sensitivity to H_2_O_2_ (i.e. a rightward shifted dose-response) compared to WT; this was despite equal constriction to the pressor agonist U-46619 in each genotype. Experiments comparing vascular reactivity in WT and KI mice after HPAH employing both tension myograph and mouse isolated lung approaches are in progress.

## Conclusion

We conclude that PKG1α plays a role in both acute hypoxic vasoconstriction as well as changes during chronic HPAH. We speculate that disulfide PKG1α levels are lowered, by chemical reduction, during acute hypoxia – potentially contributing to acute hypoxic pulmonary vasoconstriction. In contrast, during chronic hypoxia there is exacerbated PKG1α oxidation which may be protective by keeping the pulmonary arterial pressure low, and thereby reducing the afterload on the right ventricle in the setting of HPAH. Redox dead PKG1α KI mice lack this protective mechanism and therefore have exacerbated hypoxia-induced pulmonary hypertension phenotype. Interventions targeting PKG1α oxidation in the pulmonary vasculature will help to test if this mechanism truly serves to attenuate HPAH progression and consequent right heart failure.

